# CD4+ T cell cytokine responses to the DAR-901 booster vaccine in BCG-primed adults: A randomized, placebo-controlled trial

**DOI:** 10.1371/journal.pone.0217091

**Published:** 2019-05-23

**Authors:** Tereza Masonou, David A. Hokey, Timothy Lahey, Alice Halliday, Luis C. Berrocal-Almanza, Wendy F. Wieland-Alter, Robert D. Arbeit, Ajit Lalvani, C. Fordham von Reyn

**Affiliations:** 1 Tuberculosis Research Centre, Respiratory Infections Section, National Heart and Lung Institute, Imperial College London, London, United Kingdom; 2 Aeras, Rockville, MD, United States of America; 3 Larner College of Medicine, University of Vermont, Burlington, VT, United States of America; 4 Geisel School of Medicine, Hanover, NH, United States of America; 5 Tufts University School of Medicine, Boston, MA, United States of America; Colorado State University, UNITED STATES

## Abstract

**Background:**

DAR-901 is an inactivated whole cell tuberculosis booster vaccine, prepared using a new scalable, broth-grown method from the master cell bank of SRL172, a vaccine previously shown to prevent tuberculosis. This study examined whether DAR-901 (a) induces CD4+ T cell cytokine profiles previously proposed as correlates of protection and (b) has a specific vaccine-induced immunological signature compared to BCG or placebo.

**Methods:**

We analysed CD4+ T cell cytokine immune responses from 10 DAR-901 recipients, 9 BCG recipients and 9 placebo recipients from the Phase I DAR-901 MDES trial. In that study, HIV-negative, IGRA-negative participants with prior BCG immunization were randomized (double-blind) to receive three intradermal injections of DAR-901 or saline placebo or two injections of saline placebo followed by an intradermal injection of BCG. Antigen-specific functional and phenotypic CD4+ T cell responses along with effector phenotype of responder cells were measured by intracellular cytokine staining.

**Results:**

DAR-901 recipients exhibited increased DAR-901 antigen-specific polyfunctional or bifunctional T cell responses compared to baseline. Vaccine specific CD4+ IFNγ, IL2, TNFα and any cytokine responses peaked at 7 days post-dose 3. Th1 responses predominated, with most responder cells exhibiting a polyfunctional effector memory phenotype. BCG induced greater CD4+ T cell responses than placebo while the more modest DAR-901 responses did not differ from placebo. Neither DAR-901 nor BCG induced substantial or sustained Th17 /Th22 cytokine responses.

**Conclusion:**

DAR-901, a TB booster vaccine grown from the master cell bank of SRL 172 which was shown to prevent TB, induced low magnitude polyfunctional effector memory CD4+ T cell responses. DAR-901 responses were lower than those induced by BCG, a vaccine that has been shown ineffective as a booster to prevent tuberculosis disease. These results suggest that induction of higher levels of CD4+ cytokine stimulation may not be a critical or pre-requisite characteristic for candidate TB vaccine boosters.

**Trial registration:**

ClinicalTrials.gov NCT02063555.

## Introduction

Tuberculosis (TB) is the leading infectious cause of death in the world and has been targeted for eradication by 2030 [1]. An improved vaccine strategy against TB will be essential for the success of this effort. The only currently licensed vaccine for TB prevention is *Mycobacterium bovis* bacillus Calmette-Guérin (BCG). Although newer analyses indicate that BCG is highly effective against pulmonary tuberculosis when given at birth, local side effects are common, and efficacy decreases after 15–20 years [2]. Thus, TB vaccine development includes both new priming vaccines to replace BCG and vaccines to boost BCG. Development has been challenging since a new vaccine must be safe in HIV and be effective in both persons with and without latent TB [3]. Modelling studies indicate that a booster vaccine with a 10-year protective duration and 40% efficacy targeted at adolescents/adults will have a greater impact on TB epidemiology than an improved BCG prime [4].

TB vaccine development has been hampered by the lack of a vaccine-induced correlate of protection. Identification of such a correlate will necessarily involve assaying immune responses from subjects in a clinical trial in which efficacy was demonstrated. The correlate may differ for priming versus boosting vaccines and for vaccines that prevent TB infection versus vaccines that prevent TB disease [5,6]. BCG priming has been shown to prevent both TB disease and TB infection [7,8], but clinical samples are not available from these older studies. BCG boosting has been shown ineffective in the prevention of TB disease [9,10] but was recently shown to reduce the risk of TB infection in adolescents in South Africa and results from immune assays on these subjects are pending [11,12].

Two modern TB vaccine candidates have demonstrated efficacy against microbiologically-confirmed TB disease: agar-grown SRL172 inactivated whole cell booster vaccine [13] and the M72/AS01_E_ vaccine [14]. Both SRL172 and M72/AS01_E_ elicit humoral and cellular immune responses in humans [6,15]. Immune assays from the SRL172 trial did not identify a correlate of protection, and immune assays are pending on the M72/AS01_E_ trial. DAR-901 is prepared from the Master Cell Bank of agar-grown SRL172 by a new scalable, broth-grown manufacturing method. Murine studies demonstrated that, compared to a BCG booster the DAR-901 booster conferred superior protection from TB challenge [16]. A Phase 1 trial of the DAR-901 booster showed safety and immunogenicity [17] and a Phase 2b Prevention of Infection Trial is underway in Tanzania.

Proposed correlates of vaccine-induced protection consist almost entirely of one or another form of *Mycobacterium tuberculosis* (Mtb)-specific Th1 cells, producing either IFNγ, TNFα or IL2. These cells are considered an important component of anti-Mtb immunity, and are believe to function by recruiting and activating innate immune cells and restricting Mtb bacterial expansion [18]. Although these cytokines are induced by BCG [19–22], recent studies evaluating IFNγ producing T cells failed to identify this subset either as protective against Mtb or as a useful predictor of vaccine effectiveness [23–25], although this cell subset was correlated with Mtb bacterial load [25].

Polyfunctional CD4+ T cells producing IFNγ, TNFα and IL2 and bifunctional T cells expressing dual combinations of these cytokines have also been proposed as vaccine-induced correlates of protection [20,21,26–31], but there are mixed observations regarding this hypothesis. For example, while an observational study comparing BCG vaccinated versus non-vaccinated calves revealed an increased polyfunctional response correlated with protection [32], this correlation was not seen in an investigational study of rhesus macaques [33,34]. Additional studies in both vaccinated and unvaccinated cynomolgus macaques, have shown control of a TB challenge correlated with Th17/Th22 associated cytokines, IL17 and IL22 [19,35–42].

Among human trials, the H4:IC31 subunit booster vaccine induced high frequencies of CD4 T cells composed of polyfunctional or bifunctional (IL2+TNFα+) cells [30]. However, in BCG-vaccinated South African infants there was no correlation between the number of BCG-elicited polyfunctional T cells and prevention of disease [23]. In a Phase 2 trial the MVA85A subunit vaccine booster failed to confer protection against TB in healthy BCG vaccinated infants [43] despite providing heightened and durable Th1 and polyfunctional T cells responses [44,45]. There is limited clinical data addressing the role of Th17/Th22 responses.

In the present study we assayed proposed cytokine correlates of immune protection in BCG-primed subjects from the Phase 1 trial of DAR-901. We sought to determine whether previously identified correlates of protection were induced and how these responses compared to those induced by a BCG booster.

## Materials and methods

### Trial design, setting and population

**Fig 1:**
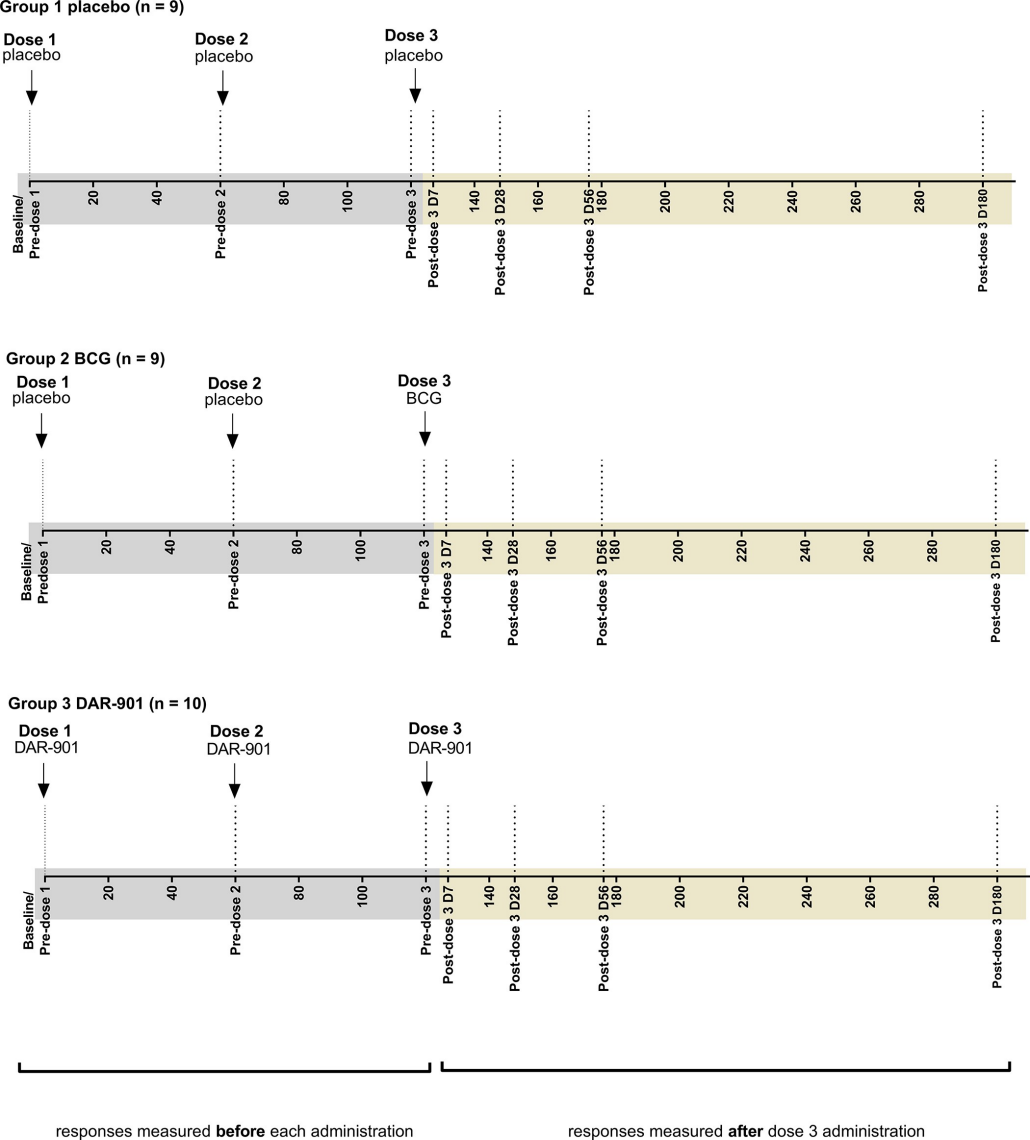
Schematic vaccination timeline of the total vaccinated subjects (n = 28) included in this study. Subjects within each group were randomized 1:1:3 to receive three doses of saline placebo-group 1 or two of saline followed by BCG (1–8 x 10^6^ CFU)-group 2, or three injections of DAR-901 7 x 10^6^ CFU / for 1 mg)-group 3. Blood samples for CD4+ T cell cytokine expression analyses were collected at baseline/pre-dose 1, pre-dose 2 and pre-dose 3 (responses measured prior to administration of respective treatment) and at 7,28,56 and 180-days after dose 3.

The DAR-901 MDES trial was a 59-subject randomized, placebo-controlled, double-blind, Phase 1 trial of a booster strategy for the prevention of tuberculosis in adult subjects aged 18–65 with a history of prior BCG immunization. Subjects included healthy IGRA negative and IGRA positive adults with and without HIV infection. The trial design has been previously described S1 Fig [17]. The present study focused on a subset of IGRA-negative subjects in the randomized dose escalation groups (cohorts A1-A3) and included a total of 28 subjects: the 10 recipients of the three 1.0 mg intradermal doses of DAR-901 (A3 cohort, the dose that has been selected for further clinical trials), the pool of 9 subjects (3 each in dose escalation cohorts A1–A3) who received 3 intradermal doses of saline placebo, and the pool of 9 subjects who received two dose of saline followed by a single intradermal dose of BCG (Fig 1). DAR-901 was produced by Aeras (Rockville, MD, USA) in compliance with Good Manufacturing Practices (7 x 10^6^ CFU / for 1 mg). Saline placebo was obtained as Sterile Saline for Injection USP, and BCG was obtained as TICE BCG (1–8 x 10^6^ CFU) (Organon Teknika, Durham, North Carolina, USA [17]. Intradermal injections of 0.1 mL were given at 0,2,4 months over the deltoid muscle alternating between right and left arms. Of note, the ten IGRA-negative subjects who received 1.0mg DAR-901 in the A3 cohort remained IGRA negative by the end of the study [17].

### Participants safety and enrolment

The DAR-901 MDES study protocol and details of the design, implementation, and safety experience in the Phase 1 DAR-901 MDES trial have been reported previously [17].

### Ethical approval

Ethical approval was obtained from the Committee for the Protection of Human subjects with ethics number #24499 at Dartmouth-Hitchcock Medical Center on 29^th^ January 2014. We obtained written informed consent from all subjects.

### Immunology assays

We collected blood for immune assays from the subjects within each group at baseline (pre-dose1), pre-dose 2, pre-dose 3 and at 7, 28, 56 and 180 days after dose 3. Vaccine-specific CD4+ T cell responses were measured from blood samples by flow cytometric intracellular staining (ICS) as described previously [46]. Thawed PBMCs resuspended in R10 medium, were rested overnight in 37°C, 5% CO_2_ incubator. Following the overnight rest, cells were counted using Guava easyCyte Flow Cytometer, cells were plated at a final concentration of 1 x 10^6^ cells per well and stimulated with medium/R10 as a negative control, staphylococcal enterotoxin B (SEB; 0.5 μg/mL) as the positive control, DAR-901 lysate or Mtb whole cell lysate at a concentration of 5 μg/mL and BCG (3 x10^5^ CFU/well). To aid in antigen processing and presentation, anti-CD28 and anti-CD49d antibodies (1 uL each per well; both from BD) were added to each well. Cells were incubated in a humidified 37 ^o^C, 5% CO_2_ incubator for 2 hrs before the addition of GolgiPlug, GolgiStop and anti-CD107a followed by an additional incubation for 6–7 hrs at 37 ^o^C and 5% CO_2_, after which the cells were placed at 2–8°C overnight. Cells were stained with Live/Dead viability dye, followed by fluorochrome-conjugated antibodies to surface markers CD4-APC, CD45RO-BV785, CCR7-BV605 then fixed and permeabilized for intracellular staining with CD3-ECD, CD8-AlexaFluor700 IFNγ-V450, TNFα-PE-Cy7, IL2-PE, IL22-APC, IL17A-PerCP-Cy5.5 and CD154-PE-Cy5. CD154 was included in the panel as a specific and sensitive marker in detecting CD4 responses [47]. Following incubation cells were washed, fixed and analysed by BD LSR flow cytometer. Analyses of flow cytometry data were performed using FlowJo v.10 using the gating template as described previously [46]. Stopping gates were set to 150,000 CD3 T cells and sufficient events (no less than 5000 CD4/CD8 T cells) were collected for analysis. Data reported are restricted to CD4+ responses as screening analyses revealed no vaccine-specific CD8+ responses using this assay.

### Statistical analysis

Data analyses were performed using GraphPad Prism v7. For immune assays, median responses pre and post challenge were compared using a Wilcoxon signed-rank test (within groups). To compare between groups our analysis included calculating the area under the curve (AUC) for responses in post-dosing time points, for immune read-outs. For non-Gaussian data, non-parametric tests were used to compared AUC values between groups using Mann Whitney U test or Kruskal-Wallis to assess differences between three groups. The threshold for the level of significance was set at a P value of <0.05.

## Results

### DAR-901 vaccine responses at 7-days post dose 3 increased compared to baseline

DAR-901 recipients demonstrated measurable increases in CD4+ T cell cytokine responses compared to baseline. DAR-901 vaccine specific CD4+ IFNγ, IL2, TNFα and any cytokine responses peaked at 7-days post-dose 3 and were significantly greater than the pre-dosing time point (see S1 Table for inter-group comparisons at each timepoint). By 180-days post-dose 3, the total CD4+ T cell cytokine production was significantly reduced from their peak levels measured at 7-days post-dose 3. However, at no time point were the responses significantly higher than the placebo group (S2 Fig). The frequency of antigen specific CD4+ T cell expression of total IL17 and total IL22 were negligible in all groups and for all stimuli.

**Fig 2:**
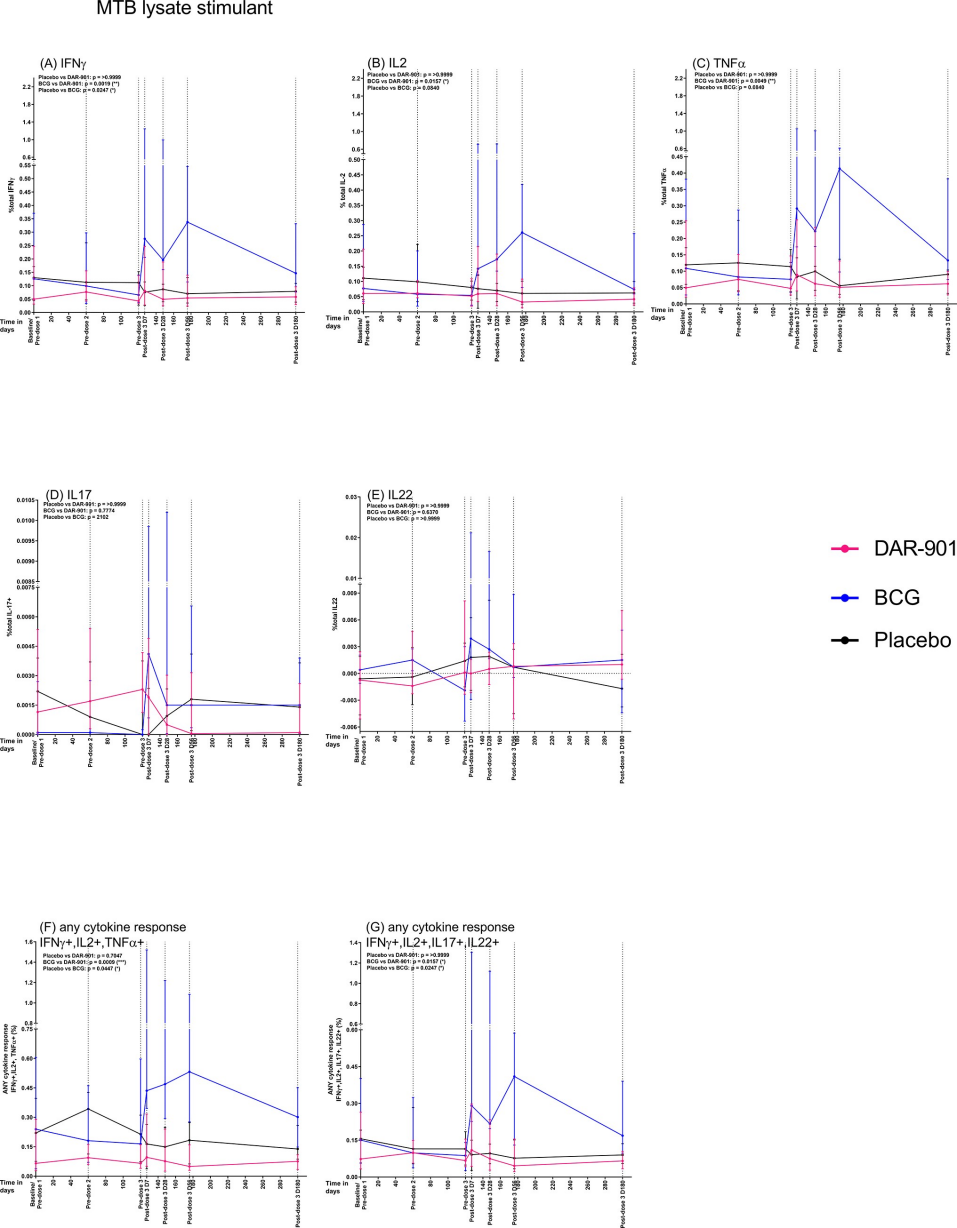
Total CD4+ T cell cytokine positive production by DAR-901, BCG and placebo vaccinated individuals in response to Mtb lysate stimulation at various study timepoints. CD4+ T cell expression of IFNγ (a), IL2 (b), TNFα (c), IL17(d), IL22 (e) and any IFNγ+, IL2+,TNFα+ cytokine (f) and any IFNγ+, IL2+,IL17+,IL22+ cytokine (g) in response to Mtb lysate stimulant in the DAR-901 (n = 10), BCG (n = 9) and Placebo (n = 9) vaccinated subject was assessed. The median CD4+ T cell cytokine responses (+IQR) are shown at each visit, after subtraction of the unstimulated levels. Treatment-specific immune responses were calculated by AUC analyses for the longitudinal immune responses for individual participants within each treatment group. The Kruskal-Wallis test (with Dunn’s post hoc test) was used to assess for differences between groups from 7-days until 180-days post dose 3 in total cytokine-producing CD4+ T cells (results shown above). Total median cytokine producing CD4+ T cell responses were compared for differences at baseline, at 7-days post dose 3 and at 180-days post dose 3) using Wilcoxon signed-rank test (results shown in S1 Table).

We evaluated whether DAR-901 vaccine resulted in the induction of Mtb-specific CD4+ T cell cytokine producing T cells with Th1 or Th17/Th22 cytokines. There were no evident differences in the frequency of Mtb-specific CD4+ T cells producing IFNγ, TNFα, IL2, IL17 and IL22 at baseline compared with post-dose time points (S1 Table, Fig 2A–2G). Compared to placebo, BCG induced modest, but statistically significantly higher frequencies of Mtb-lysate-specific CD4+ T cells producing IFNγ, TNFα and IL2 and of CD4+ T cells expressing any combination of cytokines within the Th1 and Th17 related cytokines.

### DAR-901 induces low frequencies of antigen-specific polyfunctional/bifunctional CD4+ T cells

**Fig 3:**
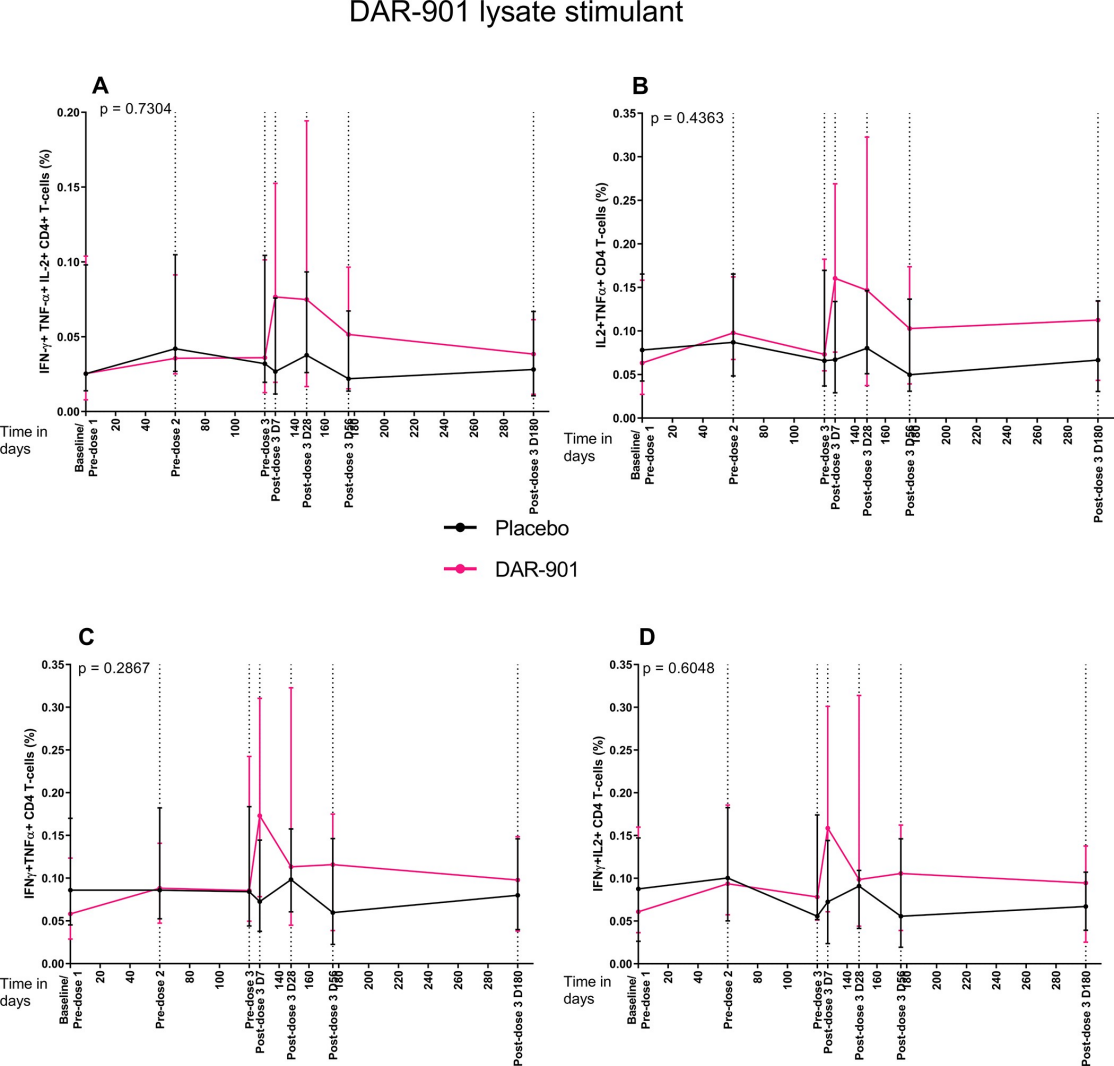
Longitudinal kinetics of DAR-901 lysate-specific CD4+ T cell responses in the DAR-901 compared to placebo vaccinated subjects. Plotted graphs represent the median for each group after subtraction of background responses, and error bars represent the IQR. Frequencies of DAR-901 lysate-specific CD4+ T cells co-expressing IFNγ, TNFα and IL2 (a) or combinations between IFNγ, TNFα, IL2 (b,c,d) at each timepoint is shown for each treatment group (DAR-901 n = 10; Placebo n = 9). For each CD4+ T cell subset the AUC was compared using Mann-Whitney U test for differences between placebo and DAR-901 treatment groups (results shown above), whilst Wilcoxon signed-rank test was used for comparison of median responses at baseline versus all post-dose 3 timepoints within the DAR-901 vaccinated group (results shown in S2 Table).

Since the DAR-901-induced CD4+ T cells to antigen re-stimulation response was only significant in the context of Th1 cytokines and any cytokine responses, we explored the induction of double and triple cytokine producing IFNγ, TNFα and IL2 CD4+ T cell responses to DAR-901 lysate (Fig 3; S3 Fig).

Among recipients of DAR-901, compared to baseline, responses to DAR-901 antigen at 7-days post-dose 3 were significantly increased, including for CD4+ bifunctional T cells producing IFNγ+ and IL2+ (p = 0.0039, Wilcoxon signed rank test) (Fig 3D; see S2 Table for inter-group comparison). At 180-dayss post dose 3, these polyfunctional and bifunctional CD4+ T cell responses had waned to frequencies similar to baseline (Fig 3A–3D; S2 Table ). Bifunctional and polyfunctional CD4+ T cell cytokine responses to Mtb lysate and DAR-901 lysate did not differ between placebo and vaccine conditions (Fig 3A–3D; S3 Fig).

### The CD4+ T cell response to DAR-901 consists predominantly of polyfunctional cells producing IFNγ+TNFα+IL2+

We characterized the proportion of DAR-901-vaccine specific CD4+ T cell cytokine responses that consisted of single cytokine responses or bifunctional and polyfunctional responses. To do this, we determined the relative proportion (median) of polyfunctional CD4+ subsets using the Boolean function within panels representing Th1 cytokines and another including Th1/Th17/Th22 cytokines.

**Fig 4:**
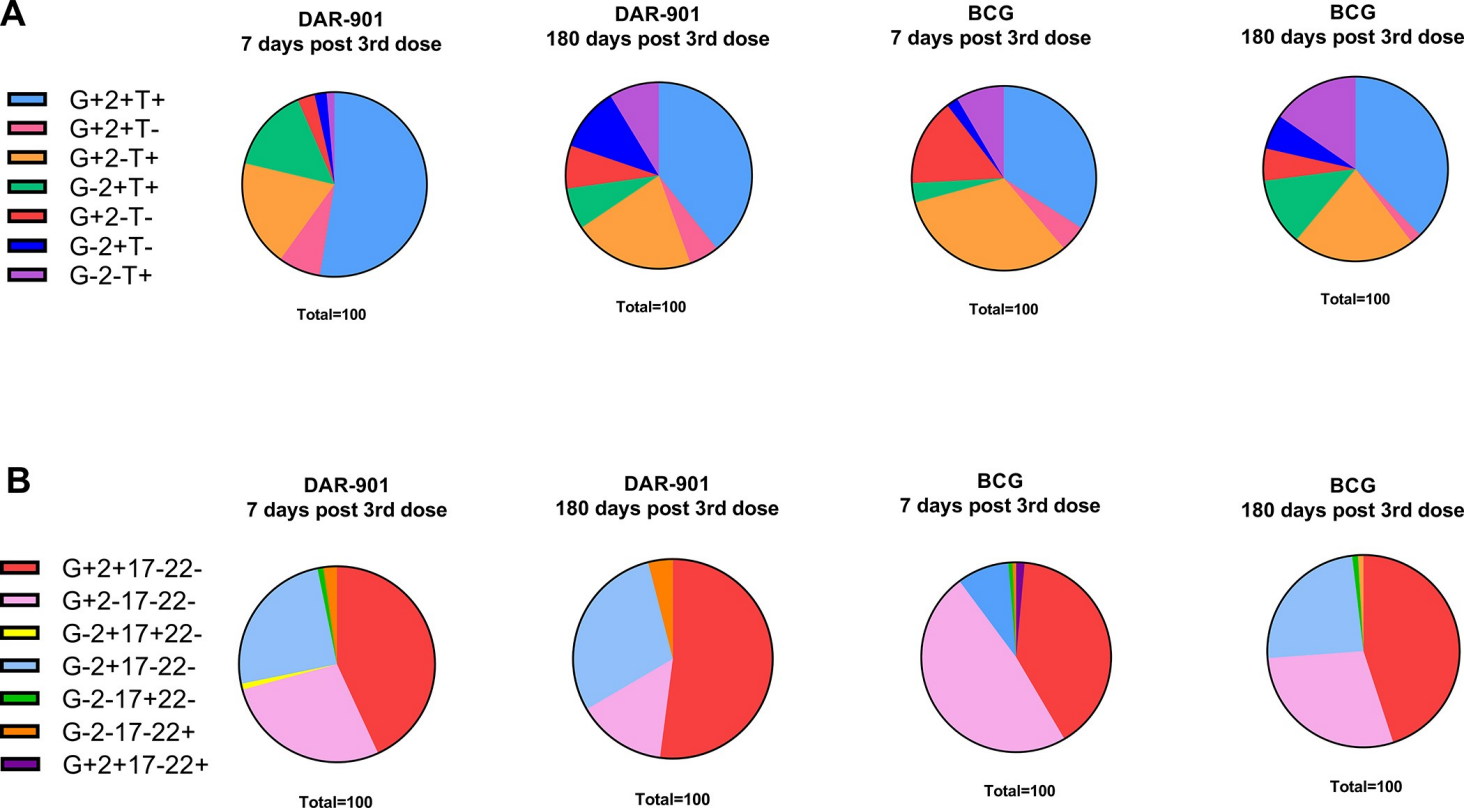
CD4+ T cell functional profiles of vaccine specific responses in DAR-901 and BCG vaccinated individuals. Side to side comparison of DAR-901 responses to DAR-901 lysate (n = 10) and BCG responses to BCG stimulant (n = 9). Pie charts represent relative proportions of respectivevaccine-specific CD4+ T cells co-producing combinations of A) IFNγ, TNFα and IL2, and B) combinations of IFNγ, IL2, IL17 and IL22 among the DAR-901 and BCG vaccinated subjects at the peak (7-days post-dose 3) and final time point (180-days post-dose 3). Frequencies of each gate were determined using Boolean gating. DAR-901 vaccine responses predominantly comprise of polyfunctional combinations of IFNγ+TNFα+IL2+ producing CD4+ T cells which were sustained until the final study point (Key: G = IFNγ, 2 = IL2, T = TNFα, 17 = IL17, 22 = IL22).

DAR-901 induced CD4+ responses consisted predominantly of polyfunctional combination of IFNγ+TNFα+IL2+ producing T cells which were sustained until the end-of-vaccine phase (Fig 4A). Although there was no substantial increase in frequencies of T cells producing Th17/Th22 related cytokines, by the end of the study a small proportion of IL22-only producing T cells is evident (Fig 4B). Consistent with previous literature, BCG responses to the BCG antigen consisted mostly of polyfunctional combinations of IFNγ+TNFα+IL2+, IFNγ+TNFα+IL2- and IFNγ+ only producing T cells. Combinations of IFNγ+TNFα+IL2+ and IFNγ+TNFα+IL2- gradually increased by the end-of-vaccine phase, whilst single-producing IFNγ+ responses decreased substantially (Fig 4A).

### DAR-901 vaccine-specific responses CD4+ T cells with a polyfunctional combination of IFNγ+TNFα+IL2+ producing CD4+ T cells were predominantly defined as terminally effector memory cells

We further compared the peak and end of study responses among DAR-901 and BCG recipients for the memory phenotype of the predominant vaccine-specific IFNγ+TNFα+IL2+ producing CD4+ T cells.

**Fig 5:**
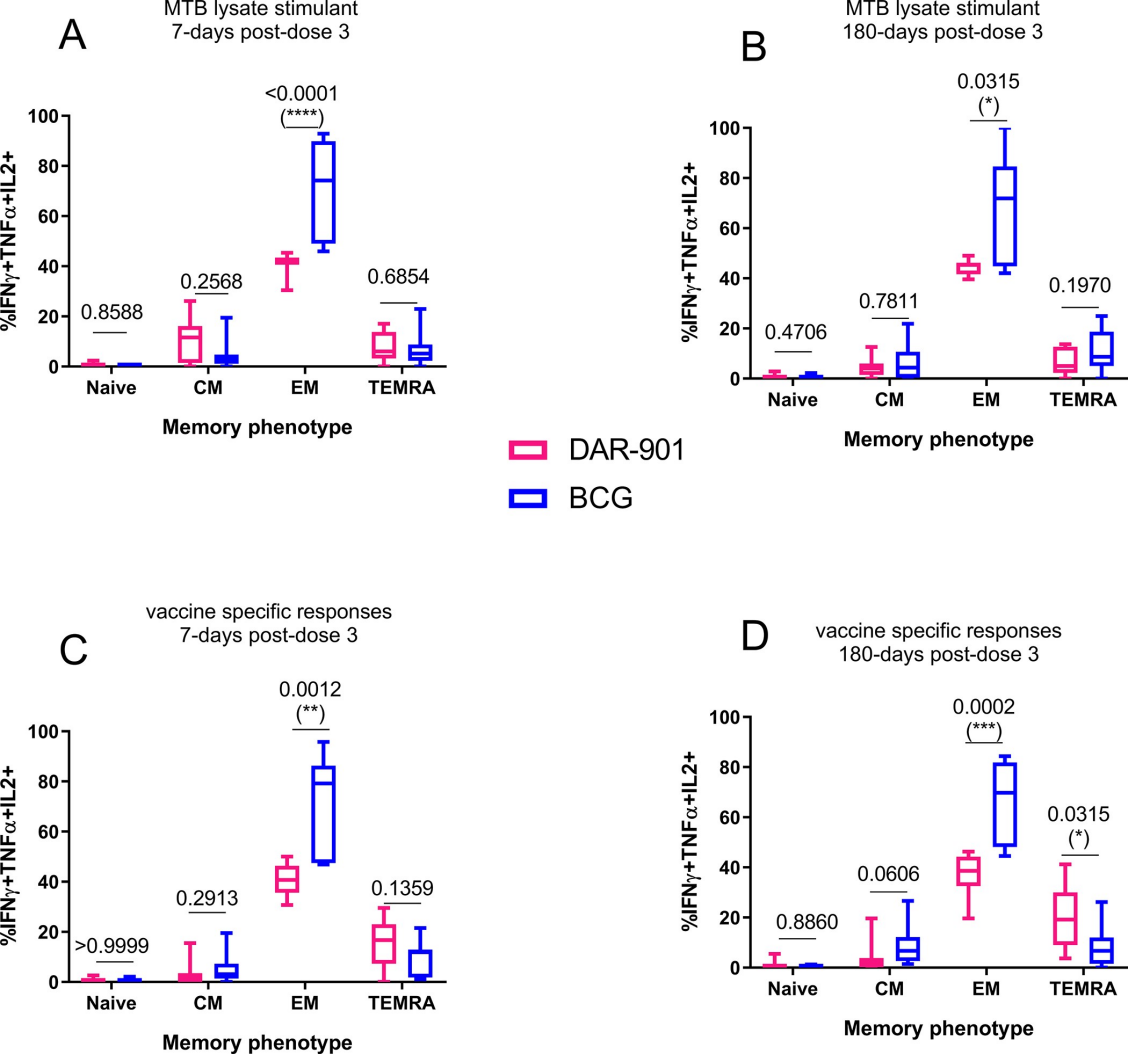
Memory phenotype of MTB and vaccine-specific CD4+ IFNγ+TNFα+IL2+ T- cell responses induced by either DAR-901 vaccine or BCG at 7 days and 180-dayss post-dose 3. Memory differentiation state was based on CD45RO versus CCR7 expression to distinguish between naïve T cells, central memory (T_CM_), effector memory (T_EM_) and T cell effector memory re-expressing CD45RA/terminally differentiated effector memory (T_EMRA_) populations. Box plots represent the T cell memory proportions comparing the frequency of vaccine-elicited CD4 memory T cells in peripheral blood in response to DAR-901 and BCG antigen re-stimulation for the respective vaccinated group. Statistical comparison using Mann-Whitney U test was used to determine the significance of differences in the proportions of memory CD4+ T cells between the two treatment groups (DAR-901 n = 10, BCG n = 9).

First, we interrogated memory differentiation state of Mtb- specific CD4+ IFNγ+TNFα+IL2+ T cells at the peak (7-days post-dose 3 and final end point of the vaccine phase (180-days post-dose 3) (Fig 5A and 5B respectively). Mtb-specific responses in both vaccines showed predominantly an effector memory phenotype both at early and end-of study timepoints. Frequencies of T_EM_ phenotype were significantly lower in the DAR-901 recipients compared to BCG recipients. At end of vaccine phase (180-days post-dose 3), but not at earlier time points (Fig 5C), DAR-901-specific polyfunctional CD4+ responder cells exhibited a terminally differentiated effector memory phenotype in DAR-901 recipients compared to BCG recipients (Fig 5D).

## Discussion

In a Phase 1 trial we found that three doses of whole cell inactivated DAR-901 immunization elicited polyfunctional Th1-type CD4+ T cell responses to the non-tuberculous mycobacterial vaccine antigen but not to Mtb lysate. DAR-901-specific responder cells exhibited predominantly an effector memory phenotype. DAR-901 represents the same inactivated non-tuberculous mycobacterial strain as SRL172 which was associated with protection from TB in an earlier Phase 3 randomized placebo-controlled double-blind clinical trial[11]. SRL172 induced IFNγ responses to the vaccine antigen and antibody to Mtb LAM but CD4+ T cell specific cytokine responses were not assessed [6,13].

Previous publications have reported that IFNγ-producing and bifunctional/polyfunctional CD4+ T cells are associated with protection from tuberculosis in experimental models [20,21,26–32]. These associations have not, however, been established in the context of a human prospective vaccine trial. Importantly, the only new candidate vaccine known to elicit human polyfunctional CD4+ T cell cytokine responses–MVA85A –did not confer protection against TB in a Phase 2 clinical trial in BCG-primed South African infants [43]. Various phenotypic and functional attributes of T cells such as recognition of an Mtb-infected cell, trafficking to the lung, and differentiation state as well as long-term memory and survival capacity been hypothesized to be central to human vaccine-mediated protection from TB, but these hypotheses have yet to be confirmed in prospective human vaccine studies.

Our data are congruent with the prior finding that DAR-901 immunization elicits IFNγ responses assessed by an ELISA platform [17]. Interestingly, we have previously found that baseline IFNγ responses to mycobacterial antigens correlate with protection from HIV-associated TB among placebo recipients in the Phase 3 trial of SRL172 whereas post-immunization IFNγ responses did not correlate with vaccine mediated protection in the same study [5,13,48]. This raises the possibility that natural TB or non-TB mycobacterial exposure together with remote BCG-induced immune protection from TB in adults may be different from booster-mediated protection from TB and thus require different methods of assessment.

Recent animal studies have suggested that Th17/Th22 cytokine production is an important and novel component of immune protection to TB [19,28,35,37,42]. In this study, we found that neither DAR-901 nor BCG immunization elicited statistically significant Th17/Th22 cytokine responses. Of note, M72/AS01_E_ induced low-level vaccine-specific Th17 responses early after vaccination [15].As with Th1 type CD4+ T cell cytokine responses, further study is required to determine whether Th17/Th22 cytokine responses are relevant to vaccine-induced immunity to TB in humans.

Strengths of this study include its prospective longitudinal follow up and assessment of a panel of multiple T cell cytokine responses to DAR-901 and other antigens. We observed clear and consistent differences in DAR-901-specific CD4+ T cell IFNγ+ and polyfunctional responses between baseline and later time points. We did not observe statistically significant differences in DAR-901-specific cytokine responses at any time point between DAR-901 and placebo recipients. This is likely because the study was underpowered to detect small differences in cytokine responses between the 10 concurrent vaccine recipients and the pool of 9 placebo recipients representing 3 subjects from the sequential three arms of this dose escalation study. DAR-901 immunization also did not induce cytokine responses to Mtb lysate when later time points were compared to baseline or in comparisons of DAR-901 recipients to placebo control. Taken together these data indicate that DAR-901 vaccination elicits low frequencies of short-lived bifunctional and polyfunctional CD4+ T cell cytokine responses to the non-tuberculous mycobacterial vaccine antigen but not Mtb lysate.

In contrast, and as expected, BCG booster recipients did exhibit greater CD4+ T cell cytokine responses to Mtb lysate at multiple time points. Since BCG booster vaccination has not been associated with improved protection from TB disease, as seen in previous clinical trials in Brazil and Malawi, [9,10,23,49,50], these data highlight the challenge of correlating detectable vaccine-related changes in immune responses to meaningful protection from TB disease. We did not observe substantial or sustained Th17/Th22 responses, either because the vaccine does not elicit such responses, or they are not well-detected in peripheral blood specimens.

Given uncertainty regarding whether large numbers of IFNγ producing T cells (Th1 response) or other CD4+ T cell cytokine responses are associated with immune protection from TB disease, it will be important to explore different types of immune responses to TB vaccine candidates [51]. Innate immune responses have not been extensively explored in TB vaccine-related clinical trials, nor in our current study. A recent study in rhesus macaques has demonstrated that neutrophil degranulation and other innate immune responses correlated with protection from TB disease in a subset of monkeys with mild disease [34]. This finding, and others, highlight the importance of examining immune responses in non-traditional cell types and perhaps even to under-investigated antigens. DAR-901 is currently being investigated for efficacy in reducing Mtb infection in a Phase 2b clinical trial among adolescents in Tanzania. We plan to explore whole blood transcriptomic responses in this trial, which will allow us to explore myeloid and lymphocyte signatures associated with susceptibility/resistance to infection.

## Conclusion

DAR-901, a TB booster vaccine grown from the master cell bank of SRL 172, a vaccine that was shown to prevent TB and to be safe and effective in a Phase 3 trial, has the potential to be an effective booster for BCG in adults and children living in tuberculosis-endemic countries. The present study has shown that DAR-901 induces low magnitude polyfunctional effector memory CD4+ T cell responses. DAR-901 responses were lower than those induced by BCG, a vaccine that has been shown ineffective as a booster to prevent tuberculosis disease. These observations suggest that induction of higher levels of CD4+ cytokine stimulation may not be a critical or pre-requisite characteristic for a candidate TB vaccine booster. A broader interrogation of immune response should be included in future clinical trials of candidate TB vaccine boosters.

## Supporting information

S1 Table Total cytokine comparison within the DAR-901 vaccinated subjects at baseline and at 7-days and 180-days post-dose 3 timepoints. Wilcoxon signed-rank test was used to assess differences between median responses.
(DOCX)

S2 Table Median combinatorial cytokine responses within the DAR-901 and placebo treatment groups vaccine-specific cytokine producing CD4+ T cells during baseline and all the post-dose 3 timepoints. Polyfunctional and bifunctional combinations of IFN*γ*, TNFα and IL2 cytokine producing T cells within the DAR-901 vaccinated group (n = 10) and placebo group (n = 9) was calculated. Wilcoxon signed rank test was used to assess differences between median responses.
(DOCX)

S1 Fig CONSORT flow chart for the DAR-901 clinical trial. The present study focused on a subset of IGRA-negative subjects in the randomized dose escalation groups (cohort A1-A3) and included a total of 28 subjects: the 10 recipients of the three 1.0mg intradermal dose of DAR-901 (A3 cohort, the dose that has been selected for further clinical trials), the pool of 9 subjects (3 each in dose escalation cohorts A1-A3) who received 3 intradermal doses of saline placebo, and the pool of 9 subjects who received two dose of saline followed by a single intradermal dose of BCG.
(DOC)

S2 Fig Total cytokine positive production by DAR-901, BCG and placebo vaccinated individuals in response to DAR-901 lysate stimulation at various study timepoints. Cytokine producing IFN*γ* (a), IL2 (b), TNFα (c), IL17 (e) and IL22 (f) T cells and a combination of any IFN*γ*+, IL2+,TNFα+ cytokine(d) and any IFN*γ*+, IL2+,IL17+,IL22+ cytokine(g) cytokine response to DAR-901 lysate stimulant in the DAR-901- (n = 10), BCG- (n = 9) and Placebo- (n = 9) vaccinated subjects was assessed (total n = 28). The median cytokine responses (+IQR) are shown at each visit, after subtraction of the unstimulated levels. Treatment specific immune responses AUC analyses are calculated for the longitudinal immune responses for individual participants for all the post-dose 3 timepoints for the respective treatment group. The Kruskal-Wallis test (with Dunn’s post hoc test) was used to assess for differences between groups in total cytokine producing T cells. Total median cytokine producing T cell responses were compared for differences at baseline, 7-days and 180-days post-dose 3 using Wilcoxon signed-rank test (results shown in S1 Table).
(TIF)

S3 Fig Longitudinal kinetics of DAR-901 Mtb-specific CD4+ T cell responses in the DAR-901 vaccinated group compared to the placebo group. Plotted graphs represent the median for each group and error bars represent the IQR, after background was subtracted. Frequencies of DAR-901 Mtb- specific CD4 T cells co-expressing IFN*γ*, TNFα and IL2 or combinations between IFN*γ*, TNFα, IL2 at each timepoint is shown for each treatment group. For each T cell subset the AUC was compared using Mann-Whitney U test for differences between placebo and DAR-901 treatment group (results shown above), whilst Wilcoxon signed-rank test was used for comparison of median responses at baseline versus all post-dose 3 timepoints within the DAR-901 vaccinated group (results shown in S2 Table).
(TIF)

S1 File CONSORT checklist. (DOC)
