# Supplementary material for: CD4+ T cell cytokine responses to the DAR-901 booster vaccine in BCG-primed adults: A randomized, placebo-controlled trial
Source: PLoS One. 2019 May 23;14(5):e0217091. doi: 10.1371/journal.pone.0217091 (PMC6532882; doi:10.1371/journal.pone.0217091)
Supplement: S1 Table — Wilcoxon signed-rank test was used to assess differences between median responses. (DOCX) [file pone.0217091.s001.docx]

**S1 Table:**

|  |  |  | **%IFNγ** | **%TNFα** | **%IL2** | **%IL17** | **%IL22** | **%any IFNγ,TNFα IL2** | **%any IFNγ,IL2, IL17,IL22** |
| --- | --- | --- | --- | --- | --- | --- | --- | --- | --- |
| **DAR-901 treatment group** | **DAR-901 lysate stimulant** | **Baseline vs  post-dose 3 D7** | 0.0078 ** | 0.0391 * | 0.0078 ** | 0.0547 | 0.3828 | 0.0039 ** | 0.0039 ** |
|  |  | **Baseline vs  post-dose 3 D180** | 0.0781 | 0.0977 | 0.1289 | 0.4258 | 0.2031 | 0.1641 | 0.0547 |
|  |  | **post-dose 3 D7 vs**  **post-dose 3 D180** | 0.0078 ** | 0.3125 | 0.0547 | 0.1094 | 0.5469 | 0.0078 ** | 0.0156 * |
|  | **MTB lysate stimulant** | **Baseline vs  post-dose 3 D7** | 0.7344 | 0.8203 | 0.7344 | 0.5703 | 0.4258 | 0.5703 | 0.6250 |
|  |  | **Baseline vs  post-dose 3 D180** | 0.8203 | 0.5703 | 0.1953 | 0.7344 | 0.7344 | 0.7344 | 0.9102 |
|  |  | **post-dose 3 D7 vs**  **post-dose 3 D180** | 0.9453 | 0.9453 | 0.9453 | 0.2188 | 0.7109 | 0.1953 | 0.4609 |
