# Supplementary material for: CD4+ T cell cytokine responses to the DAR-901 booster vaccine in BCG-primed adults: A randomized, placebo-controlled trial
Source: PLoS One. 2019 May 23;14(5):e0217091. doi: 10.1371/journal.pone.0217091 (PMC6532882; doi:10.1371/journal.pone.0217091)
Supplement: S2 Table — Polyfunctional and bifunctional combinations of IFNγ, TNFα and IL2 cytokine producing T cells within the DAR-901 vaccinated group (n = 10) and placebo group (n = 9) was calculated. Wilcoxon signed rank test was used to assess differences between median responses. (DOCX) [file pone.0217091.s002.docx]

**S2 Table:**

|  |  |  | **%IFNγ+ TNFα+ IL2+** | **%IL2+TNFα+** | **%IFNγ+ TNFα+** | **%IFNγ+IL2+** |
| --- | --- | --- | --- | --- | --- | --- |
| **DAR-901 treatment group** | **DAR-901 lysate stimulant** | **Baseline vs   post-dose 3 D7** | 0.0195 * | 0.0117 * | 0.0195 * | 0.0039 ** |
|  |  | **Baseline vs**  **post-dose 3 D28** | 0.1484 | 0.1484 | 0.0781 | 0.1953 |
|  |  | **Baseline vs  post-dose 3 D56** | 0.7422 | 0.5469 | 0.3125 | 0.8438 |
|  |  | **Baseline vs**  **post-dose 3 D180** | 0.1641 | 0.1289 | 0.1641 | 0.0977 |
| **Placebo treatment group** | **DAR-901 lysate stimulant** | **Baseline vs   post-dose 3 D7** | 0.0547 | 0.0273 * | 0.0742 | 0.0977 |
|  |  | **Baseline vs**  **post-dose 3 D28** | 0.7422 | 0.7422 | 0.8438 | >0.9999 |
|  |  | **Baseline vs  post-dose 3 D56** | 0.2031 | 0.2500 | 0.2031 | 0.3594 |
|  |  | **Baseline vs**  **post-dose 3 D180** | 0.1719 | 0.2031 | 0.4961 | 0.6523 |
