# Supplementary material for: CD4+ T cell cytokine responses to the DAR-901 booster vaccine in BCG-primed adults: A randomized, placebo-controlled trial
Source: PLoS One. 2019 May 23;14(5):e0217091. doi: 10.1371/journal.pone.0217091 (PMC6532882; doi:10.1371/journal.pone.0217091)
Supplement: S1 Fig — The present study focused on a subset of IGRA-negative subjects in the randomized dose escalation groups (cohort A1-A3) and included a total of 28 subjects: the 10 recipients of the three 1.0mg intradermal dose of DAR-901 (A3 cohort, the dose that has been selected for further clinical trials), the pool of 9 subjects (3 each in dose escalation cohorts A1-A3) who received 3 intradermal doses of saline placebo, and the pool of 9 subjects who received two dose of saline followed by a single intradermal dose of BCG. (DOC) [file pone.0217091.s003.doc]

**
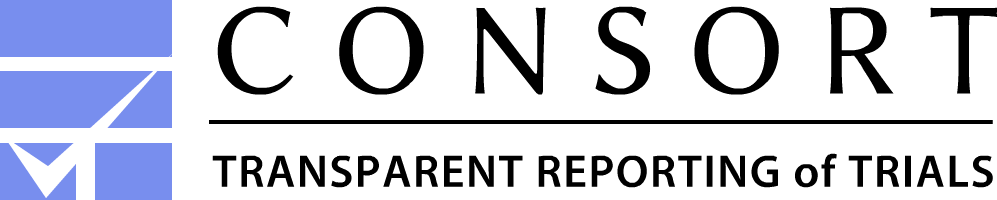
**

**CONSORT 2010 Flow Diagram**

Randomized (n= 49)
Immunized (n=48)

***Cohorts A1, A2, A3
(randomized)***

**Allocation**

**Analysis**

**Enrollment**

Assessed for eligibility (n= 66)

Excluded (n=18)

♦  IGRA positives (n= 9)

♦  Abnormalities (n= 4)

♦  Other reasons (n= 4)

Placebo/BCG
(n=3)

DAR-901 0.1mg (n=10)

A1 (n=16)

Total participants included in this study n=28

A2 (n=16)

A3 (n=16)

DAR-901
0.3 mg (n=10)

DAR-901
1.0 mg (n=10)

Placebo
(n=3)

Placebo
(n=3)

Placebo/BCG
(n=3)

Placebo/BCG

(n=3)

Placebo
 (n=3)

***Cohorts A4, B1, B2
(open label)***

**Allocation**

**Enrollment**

**Not included in this study**

Assessed for eligibility (n= 12)

Excluded (n=1)

♦  Prior carcinoma

DAR-901
1.0mg (n=5)

A1 (n=5)

Enrolled (n=11)

B2 (n=1)

B1 (n=5)

DAR-901
1.0 mg (n=1)

DAR-901
1.0 mg (n=5)

***HIV -ve
IGRA +ve***

***HIV +ve
IGRA -ve***

***HIV +ve
IGRA +ve***
